# Educational Model and Prevention on Prediabetes: A Systematic Review

**DOI:** 10.2174/0115733998275518231006074504

**Published:** 2024-02-14

**Authors:** Rina Amelia, Juliandi Harahap, Zulham, Isti Ilmiati Fujiati, Hendri Wijaya

**Affiliations:** 1 Department of Community Medicine/Public Health Sumatera Utara, Faculty of Medicine, Universitas Sumatera Utara, Utara, Indonesia;; 2 Department of Histology, Faculty of Medicine, Universitas Sumatera Utara, Medan, Indonesia;; 3 Department of Paediatics, Faculty of Medicine, Universitas Sumatera Utara, H. Adam Malik General Hospital, Madan, Indonesia

**Keywords:** Prediabetes, educational model, lifestyle modification, prophylaxis, glycemic control, diabetes mellitus (DM)

## Abstract

**Background:**

Prediabetes is a reversible condition before the onset of Type 2 Diabetes Mellitus. Untreated condition of prediabetes will develop into diabetes and its complications. The prevalence of prediabetes has been emerging worldwide and has a considerable socioeconomic impact. The current study reviews the roles of early detection, educational models, life modification, and prophylaxis of individuals with prediabetes in preventing the progression of prediabetes into Type 2 Diabetes Mellitus and complications in the future.

**Methods:**

This study included published articles from several electronic databases. The obtained articles were limited to March 2023. Articles that were not open access and not in Indonesian or English were excluded. The protocol for this study used the PRISMA (Preferred Reporting Items for Systematic Reviews and Meta-Analyses) 2020.

**Results:**

Of 39627 articles, 39601 were excluded due to duplication and did not meet the eligibility criteria. At the final, there were 26 articles that were eligible for systematic review.

**Conclusion:**

Prevention of the development of prediabetes into diabetes is essential. A comprehensive understanding and training on intensive lifestyle modification protocols from local and national experts in diabetes prevention through digital-based education models and linguistically and culturally approach can be considered. Intensive lifestyle modification and pharmacological approaches may improve the outcome. Regular monitoring of glycemic control is also important for early diagnosis of diabetes, especially in patients with special conditions.

## 1 INTRODUCTION

Diabetes mellitus (DM) is one of the most common metabolic diseases which is characterized by hyperglycemia due to dysfunction of insulin secretion, insulin resistance, or both. Type 2 Diabetes Mellitus (T2DM) is a form of DM whereby the tissue is resistant to insulin [1]. The long-term state of hyperglycemias in diabetes causes long-term damage and failure of various organs, especially the eyes, nerves, kidneys, blood vessels, and heart [2]. The diabetes prevalence among the age group 20-79 years in 2021 is 10.5%. It affects 536.6 million people globally, and over half of a billion people have diabetes worldwide. By 2045, the diabetes prevalence is estimated to rise to 12.2% and will affect 783.2 million adults. DM is reported to be higher in urban than rural and affects men and women equally. The prevalence was also highest in those aged 75-79 years. Healthcare expenditure for people with diabetes is expected nearly 1,054 billion USD by 2045 [3].

DM is currently one of the major public health problems worldwide [3, 4]. In Indonesia, the prevalence of T2DM is rapidly increasing annually. According to RISKESDAS (Basic Health Research Indonesia), the prevalence of T2DM among the age group older than 14 years old increased by 0.5% from 2013 to 2018. However, the prevalence of T2DM according to the blood glucose test increased from 6.9% in 2013 to 8.5% in 2018. About 25% of diabetics are aware that they had diabetes [5]. Medical costs for the treatment of T2DM with complications are twice as high as for T2DM without complications [6].

Prediabetes is the term used for individuals with impaired fasting plasma glucose (IFG) and/or impaired glucose tolerance (IGT) and demonstrates the condition before the onset of T2DM. IFG and IGT are usually associated with risk factors for diabetes such as obesity (abdominal or visceral obesity), hypertension, and dyslipidemia with high triglycerides and/or low HDL cholesterol. According to American Diabetes Association (ADA) on diagnosis, prediabetes defined as fasting plasma glucose (FPG) levels 100 mg/dL (5.6 mmol/L) to 125 mg/dL (6.9 mmol/L) or 2-hours plasma glucose in the 75-gram oral glucose tolerance test (OGTT) levels 140 mg/dL (7.8 mmol/L) to 199 mg/dL (11.0 mmol/L) or HbA1C levels 5.7 to 6.4% [7]. Those criteria are sufficient to confirm prediabetes. The prevalence of prediabetes reported in the literature varies widely due to the diagnostic criteria used, the choice of test and the populations being studied [8]. The global prevalence of prediabetes according to IGT test was estimated to affect 352.1 million adults in 2017. According to the International Diabetes Federation (IDF), we estimated the IGT prevalence at 7.5% in both men and women. The prevalence is anticipated to increase to 587 million adults by 2045 [8, 9]. The vast majority of people with prediabetes settle in low- and middle-income countries (LMICs). In the United States, the prevalence of prediabetes among the adult population aged 20 or older at 2.5% (all criteria were diagnosed) and 51.3% (any of one of the criteria was diagnosed). Larger surveys in China have shown the prevalence of prediabetes on any one of the three glycemic tests varied from 36% in one study to 50.1% in another study. The North America and Caribbean regions have the highest IGT prevalence and the European region has the lowest IGT prevalence [9]. In reality, the prevalence of prediabetes is greater than the prevalence of diabetes. In Iran, the prevalence of prediabetes and DM was 18% and 10%, respectively [10]. The prevalence of isolated IFG is higher in Caucasians. Otherwise, the prevalence of isolated IGT and combining both IFG and IGT is higher in Asians [11].

Prediabetes is considered a critical phase where the condition is reversible and increases the risk of developing T2DM in the future. People with prediabetes often do not realize that their blood glucose levels have increased due to the absence of signs and symptoms until diabetes is diagnosed [1, 7]. It may occur due to a lack of knowledge about prediabetes and the prevention from prediabetes to diabetes. This study aims to review early detection, educational models, life modification, and prophylaxis in individuals with prediabetes to prevent progression to T2DM and complications in the future.

## 2 MATERIALS AND METHODS

The method of this study used a systematic review approach. The collection and review of articles were obtained through several electronic databases, such as PubMed and Google Scholar using the keywords Prediabetes, DM, Diabetes mellitus type 2, T2DM, Screening, Educational Model, Treatment, Metformin, Monitoring, and Prevention. The articles obtained were limited to March 2023 and selected based on the inclusion and exclusion criteria in order to obtain relevant articles. The articles are included in Indonesian and English and open access. Moreover, the selected article’s design was cross-sectional, case control, cohort, randomized controlled trial (RCT), and case report. The literature review used the PRISMA 2020.

## 3 RESULTS

**Fig. (1):**
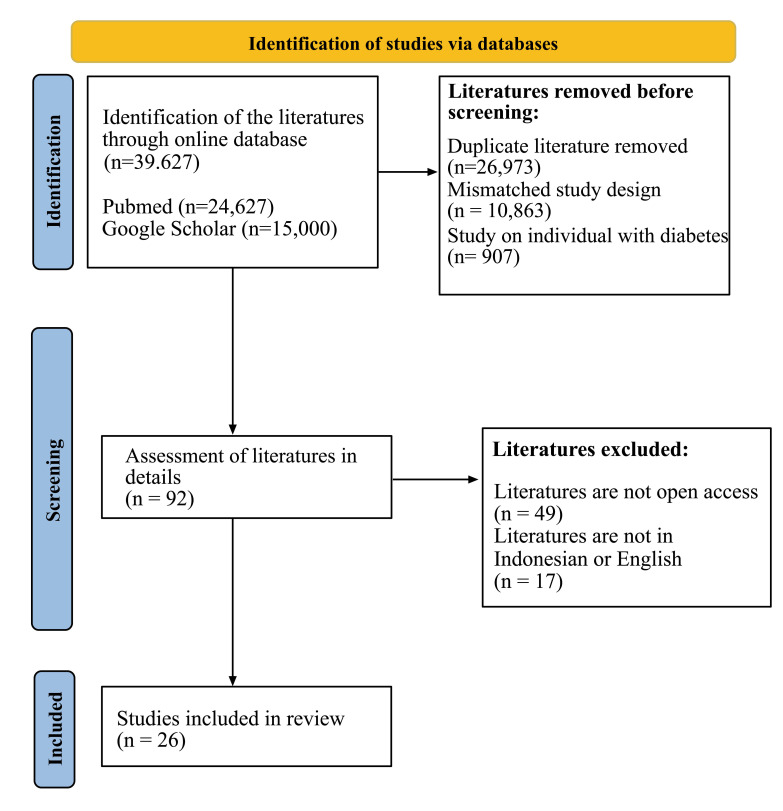
The study flow selection using PRISMA 2020.

Of the 39627 articles that were initially traced, 24627 were obtained from Pubmed and 15000 from Google Scholar. Then, 39535 articles were excluded, 26973 articles due to duplication, of them were duplicated, 10863 articles due to mismatched study design and 907 articles due to the subjects were individuals with diabetes. Ninety-two full text articles were assessed for their eligibility to be analyzed in detail, forty-nine of them were excluded because the articles were not open-access, and seventeen articles were not in Indonesian or English. At the final stage, there were 26 articles that were considered to meet the inclusion criteria (Fig. **1**).

Fig. **(1)**, of the 26 eligible articles in this systematic review, the study design of 4 articles was the cohort, 2 articles were cross-sectional and 20 articles were randomized control trials. A total of 11 articles discussed the educational model, 16 articles discussed lifestyle modification, and 11 articles discussed prophylaxis of an individual with prediabetes to prevent the development of T2DM.

## 4 DISCUSSION

This systematic review has summarized the results of 26 studies on the prevention of the incidence of T2DM progression in individuals with prediabetes. Final data were obtained to review the prevention of progression prediabetes in terms of early detection, pharmacological interventions, and non-pharmacological interventions. Non-pharmacological intervention consists an educational models and life modification. However, prophylaxis of the progression of prediabetes to T2DM may require pharmacological intervention.

### 4.1 Early Detection

Individuals with prediabetes are often asymptomatic. Testing to assess prediabetes should be considered in adults of any age who have additional risk factors for diabetes:

- Overweight or obese (BMI ≥25 kg/m^2^ or ≥23 kg/m^2^ in Asian Americans).
- Physical inactivity.
- First-degree relative with diabetes.
- High-risk race/ethnicity (*e.g*., African American, Pacific Islander, Latino, Native American, and Asian American).
- Hypertension (≥140/90 mmHg) or on therapy for hypertension.
- HDL cholesterol level <35 mg/dL (0.90 mmol/L) and/or a triglyceride level >250 mg/dL (2.82 mmol/L).
- HbA1C ≥5.7%, IGT, or IFG on previous testing.
- History of cardiovascular disease.
- Other clinical conditions associated with insulin resistance (*e.g*., severe obesity, acanthosis nigricans).
- Women who delivered a baby weighing >4 kg or were diagnosed with gestational DM.
- Women with polycystic ovary syndrome [7].

The FPG, 2-h-PG after 75 g OGTT, and HbA1C are appropriate to be used in diagnosing prediabetes. Several studies demonstrated that HbA1C was a strong predictor for the development of T2DM. HbA1C reflects the cumulative glycemic history of the preceding two to three months period [7, 12]. The prediabetes usually have the HbA1C levels at 5.7% to 6.4%. The International HbA1C Consensus Committee has recommended that the HbA1C level have to be reported in terms of System International (SI) units. The SI unit of HbA1C levels were millimoles per mole with no decimal places. It allows for avoiding any confusion between HbA1C levels and FPG levels as millimoles per Liter (Table **1**) [12].

HbA1C does not only provide chronic hyperglycemia which is a reliable measure but also correlates with the long-term complication of T2DM as well. Elevated HbA1C level has been determined as a significant risk factor for cardiovascular and cerebrovascular diseases. Thus, a single HbA1C has been identified as a reliable biomarker for the diagnosis and prognosis of diabetes [12]. It is recommended as a routine screening test to assess prediabetes, especially in an individual with one or more additional risk factors.

### 4.2 Educational Model

Various studies have been conducted to discover suitable educational models for individuals with prediabetes. A low-cost community-based peer-supported lifestyle intervention through The Kerala Diabetes Prevention Program in Kerala State, India, resulted in a nonsignificant reduction in T2DM incidence at 2 years [13]. Furthermore, another study assessing the effectiveness of The Let’s Prevent T2DM Program, a low-resource group based-structured-education within primary care in the United Kingdom also resulted in a nonsignificant reduction to the risk of T2DM, in spite of that, this program contributed modest benefits to biomedical, lifestyle and psychosocial [14]. RCT studies in rural China evaluated the effectiveness of one year’s synthetic intervention in preventing T2DM among prediabetes elderly. This synthetic intervention model was formulated through consultation with several experts, focus group discussions including lifestyle education for 3 months, lifestyle intervention for 3 months, training for the Self-Monitoring of Blood Glucose for 3 months and setting up a Help Other Group. As a result, it can reduce the incidence of T2DM and improve the metabolic outcome [15]. Another RCT study among Danish adults with prediabetes also demonstrated the reduced effect on weight, waist circumference, and systolic blood pressure one year after a brief history-based health promotion intervention was implemented [16].

The digital-based educational model discussed in some articles included in this systematic review has different results from educational model-based peer-support or structured education such as The Kerala Diabetes Prevention Program and The Let’s Prevent T2DM Program. There are some RCT studies exploring that text messages *via* SMS can lead to an effective and acceptable method to provide advice and support lifestyle modification. Thus, it reduces the risk of development of prediabetes and has significant potential for widespread dissemination and impact [17-22]. Text message intervention can lead to clinically significant weight loss among prediabetics, reducing 5.05% onset of T2DM and cost-saving [17, 21]. In addition, high-frequency telephone-delivered lifestyle modification support also could effectively prevent the progression from prediabetes to T2DM in primary healthcare settings [22]. Linguistics and culture can affect the effectiveness of the educational models. In the study conducted by Yeh *et al*. (2016), a significant weight loss and improvement in HbA1C concentration was found in the Diabetes Prevention Program among Chinese immigrants with prediabetes living in New York [23]. Based on these explanations, the digital-based education model and linguistical and cultural approach can be considered as one of the nonpharmacological interventions particularly considering the growing demand for telemedicine in preventive healthcare services.

### 4.3 Life Modification

Various studies have recommended a genetic disposition for insulin insensitivity. There are certain epigenetic risk factors, such as obesity, lack of physical exercise, and physically inactive lifestyle exacerbating insulin resistance [1]. Lifestyle intervention objects the risk factors in terms of regular dietary advice, physical activity instruction, and weight loss. A most recent overview of systematic reviews discussed the effectiveness of dietary, physical activity, and weight loss in preventing comorbidity and mortality of T2DM [24]. Management of a healthy lifestyle diet such as reduces total and saturated fat intake and enhances the consumption of a nutritious diet that contains food with a low glycemic index, such as cereal fiber or a mixture of whole grain and bran. The result study conducted in Vietnam showed an association between improvement in HDL cholesterol and anthropometric parameters such as waist circumference, waist-to-hip ratio, BMI, and weight. Moreover, reducing the consumption of sugar-containing beverages significantly reduces the risk of progression to prediabetes. For instance, drinking more than one sugar-containing beverage per day has a 26% higher risk of developing T2DM compared to drinking less than one sugar-containing beverage per month [1, 25].

Physical exercise improves β-cell function and insulin sensitivity among pre-diabetics as well as diabetics by increasing free fatty acid oxidation, enhancing skeletal muscle mitochondrial function, reducing lipotoxicity in skeletal muscles and liver and increasing the serum level of adiponectin. It also acts as a physiological stressor which increases glucose uptake by muscle cells and activates GLUT-4. Activation of GLUT-4 allows glucose to enter the cell [1]. A study conducted in 2016 found the physical activity goal of 150 min per week was associated with reduction of body weight by 4.2%. It showed a mean weight loss of 5%-7% among prediabetics and contributed to a 58% reduction in progression in individuals with prediabetes [26]. Other studies also demonstrated a significant result in reducing weight and HbA1C and improving the mean score of health behaviours, exercise aspects, stress management, and capillary plasma glucose. Moderate-intensity exercise consisting of music helps to motivate participants to perform more exercise [27, 28].

Lifestyle interventions including a healthy diet accompanied by a significant increase in physical activity can prevent the risk of T2DM by 40% - 47% for 2 years [29]. Furthermore, the cost of T2DM cases prevented from various study analysis demonstrated that lifestyle intervention resulting in an equivalent reduction in health care costs [24]. Thus, implementing long-term lifestyle modification is effective in delaying progression to T2DM, safe and can reduction in healthcare costs, especially in the LMICs.

### 4.4 Pharmacological Interventions

There is some evidence showing oral anti-diabetics (OAD) as one of the options to prevent the progression of prediabetes. ADA recommended metformin as an option for T2DM prevention. Metformin is effective, safe, convenient, and cost-effective, especially in patients younger than 60 years and BMI >35 Kg/m^2^, FBG ≥ 110 mg/dL (6.1 mmol/L) or HbA1C ≥6%, and women with a history of gestational diabetes [30]. Women with prior gestational diabetes have a seven-fold increased risk of developing T2DM. Consumption of metformin 850 mg twice daily reduced the incidence of T2DM by 20% [31]. The relative risk reduction was 20.8% with prediabetics who adhered to the consumption of metformin for 10 years [32].

Metformin has some side effects, such as gastrointestinal disorders and B12 deficiency. Moreover, lactic acidosis, a rare side effect, could occur with the chronic use of metformin, especially in an individual with a decline in renal function [30]. Besides that, there is various studies discussing that new OAD including Sodium-Glucose Transport Protein 2 Inhibitors (SGLT2i) and Glucagon-Like Peptide-1 Receptor Antagonist (GLP-1 RA) may have advantageous effects on preventing the progression of prediabetes. The use of dapaglifozin in patients with heart failure reduces the incidence of new T2DM by 32% [30]. Intensive lifestyle intervention conducted on Hispanic women in the United States demonstrated a significantly greater mean weight loss of about 4 kg than metformin 0.9 kg and weight gain of about 0.8 kg in standard care patients [33]. The combination of intensive lifestyle intervention and OAD may improve their effect on preventing the progression of prediabetes to diabetes. However, further research is needed to investigate it.

### 4.5 Laboratory Monitoring

The use of monitoring laboratories in early detection is expected to prevent the development of T2DM from prediabetes and reduce the risk of its complications. There are several tests that can identify the progression of prediabetes, including homeostasis model assessment of insulin resistance (HOMA-IR) and HbA1C. The HOMA-IR is a convenient and inexpensive test to estimate insulin resistance. HOMA-IR correlated with various metabolic parameters including FPG, HbA1C, and blood pressure. In the Chinese population, the HOMA-IR cut-off discriminated prediabetes from normal glucose test was 1.4 [34]. Further study is needed to investigate the HOMA-IR cut-offs in other populations. HbA1C has not only been identified as a reliable biomarkers for long-term glycemic control, but has also contributed to cardiovascular disease risk assessment. HbA1C may better predict incident cardiovascular disease than FPG and 2-h-PG. Elevated HbA1C in people with prediabetes has an 80% greater cardiovascular disease risk [35].

Hemoglobin’s life span in red blood cells is approximately 90-120 days. Therefore, a non-immunoglobulin serum protein has a much lower half-life, approximately 14-21 days. HbA1C is associated with hemoglobin life span and characteristics [36]. Thus, HbA1C accuracy can decrease in certain conditions, such as anemia and end-stage renal disease [37]. Therefore, monitoring glycemic control using glycated albumin and fructosamine can be considered. Glycated albumin (GA) is a ketoamine formed *via* a non-enzymatic glycation of serum albumin. GA has a half-life of approximately 20 days. It can be used to assess glycemic control in the short term. Various studies have demonstrated that GA has a strong positive correlation to assess glycemic control as well as an additional test in detecting patients with diabetes [36, 37]. Besides that, using GA as the sole T2DM diagnostic test should be interpreted with caution [38]. GA levels are related to the total albumin levels, which affect patients with protein-losing states such as hepatic cirrhosis, nephrotic syndrome, and thyroid disease. All of these conditions induce hypoalbuminemia. However, fructosamine levels are not generally correlated with albumin or total protein concentration. Hypoproteinemia such as pregnancy or malnutrition are more likely to affect fructosamine levels. The concentration of fructosamine is influenced by the levels of immunoglobulins, especially IgA [37]. Regular monitoring of glycemic control is expected to prevent the progression of prediabetes and act as an early diagnosis of T2DM even in patients with special conditions.

## CONCLUSION

The incidence of T2DM developing from diabetes has been increasing. This condition can cause global health problems. Intensive lifestyle modification and pharmacological approaches may prevent the development of diabetes from prediabetes. A comprehensive understanding and training on intensive lifestyle modification protocols from local and national experts in diabetes prevention through digital-based education models and linguistic and cultural approaches can be considered. Regular monitoring of glycemic control is also important to perform early diagnosis of diabetes, especially in patients with special conditions.

## Figures and Tables

**Table 1:** The conversion of HbA1C and blood glucose.

**Blood Glucose**		**Interpretation**	**HbA1C**	
**mmol/L**	**mg/dL**		**%**	**mmol/mol**
5.4	97	Normal	5	31
7.0	126		6	42
8.6	155	Prediabetes	7	53
10.2	184	Diabetes	8	64
11.8	212	Diabetes	9	75
13.4	241		10	86
14.9	268	Diabetes	11	97
16.5	297		12	108

**Note:** [12].

## Data Availability

The data and supportive information are available within the article.

## LIST OF ABBREVIATIONS

ADA: American Diabetes Association
BMI: Body Mass Index
DM: Diabetes Mellitus
FPG: Fasting Plasma Glucose
GA: Glycated Albumin
GLP-1 RA: Glucagon-Like Peptide-1 Receptor Antagonist
GLUT 4: Glucose Transporter Type 4
HbA1C: Glycated Hemoglobin A1C
HDL: High-Density Lipoprotein
HOMA-IR: Homeostasis Model Assessment of Insulin Resistance
IDF: International Diabetes Federation
IFG: Impaired Fasting Plasma Glucose
IGT: Impaired Glucose Tolerance
LMICs: Low- and Middle- Income Countries
OAD: Oral Anti Diabetic
OGTT: Oral Glucose Tolerance Test
PG: Plasma Glucose
PRISMA: Preferred Reporting Items for Systematic Reviews and Meta-Analyses
RCT: Randomized Controlled Trial
SGLT2i: Sodium-Glucose Transport Protein 2 Inhibitors
SI: System International
T2DM: Type 2 Diabetes Mellitus

## SUPPLEMENTARY MATERIAL

PRISMA checklist is available as supplementary material on the publisher’s website along with the published article. Supplementary material is available on the publisher’s website along with the published article.
